# The free energy landscape of the oncogene protein E7 of human papillomavirus type 16 reveals a complex interplay between ordered and disordered regions

**DOI:** 10.1038/s41598-019-41925-4

**Published:** 2019-04-09

**Authors:** Predrag Kukic, Giuseppe Mattia Lo Piccolo, Marcela O. Nogueira, Dmitri I. Svergun, Michele Vendruscolo, Isabella C. Felli, Roberta Pierattelli

**Affiliations:** 1 Department of Chemistry, University of Cambridge, Cambridge, CB2 1EW UK; 2 Magnetic Resonance Center (CERM), University of Florence, 50019 Sesto Fiorentino, Italy; 3 European Molecular Biology Laboratory, Hamburg Unit, Notkestrasse 85, D-22603, Hamburg, Germany; 4 Department of Chemistry “Ugo Schiff”, University of Florence, 50019 Sesto Fiorentino, Italy

## Abstract

When present, structural disorder makes it very challenging to characterise the conformational properties of proteins. This is particularly the case of proteins, such as the oncogene protein E7 of human papillomavirus type 16, which contain both ordered and disordered domains, and that can populate monomeric and oligomeric states under physiological conditions. Nuclear magnetic resonance (NMR) spectroscopy is emerging as a powerful method to study these complex systems, most notably in combination with molecular dynamics simulations. Here we use NMR chemical shifts and residual dipolar couplings as structural restraints in replica-averaged molecular dynamics simulations to determine the free energy landscape of E7. This landscape reveals a complex interplay between a folded but highly dynamical C-terminal domain and a disordered N-terminal domain that forms transient secondary and tertiary structures, as well as an equilibrium between a high-populated (98%) dimeric state and a low-populated (2%) monomeric state. These results provide compelling evidence of the complex conformational heterogeneity associated with the behaviour and interactions of this disordered protein associated with disease.

## Introduction

Papillomavirus-related infections represent an ubiquitous threat because of their causative link to a range of different types of cancer, including in particular cervical cancer^1,2^. By studying the molecular processes underlying the ability of the virus to infect its hosts, it has been recognised that the oncogenic potential of two high-risk strains, human papillomavirus types 16 (HPV-16) and 18 (HPV-18), rely on the expression of the intrinsically disordered proteins E6 and E7, which are necessary for the induction and maintenance of the transformed phenotype^2,3^. Although interactions of E6 and E7 with a wide variety of cellular partners have been identified, the detailed molecular mechanisms of these interactions are not yet clearly understood, primarily because of the intrinsically disordered nature of the two proteins^4–11^.

HPV-16 E7 is a 98-residue protein with three conserved regions (CRs). Its N-terminal half contains two CRs, namely CR1 (residues 1–15) and CR2 (residues 16–40), which show a high propensity to be disordered^9–12^ and interact with a wide variety of other proteins^13,14^. Among them, the interaction of CR2 with retinoblastoma (Rb), a tumour-suppressor protein dysfunctional in many cancer types, via the LXCXE motif is particularly studied due to its ability to disrupt Rb-mediated control of cell proliferation^15,16^. Another often studied motif of CR2 is a consensus casein kinase II (CKII) phosphorylation site, which is among many contributors to the transforming activity of E7^17^. This promiscuity of the N-terminal half of E7 is facilitated by its intrinsic flexibility induced by richness of acidic residues that translate into a theoretical pI of about 3.5^11^. Despite the absence of a compact-cooperative structure, the N-terminal half of E7 (E7N) is often considered as a *bona fide* domain^9^.

The C-terminal half of E7 (E7C) contains another conserved region, CR3 (residues 41–98), that binds one zinc ion (Zn^2+^) by two highly conserved CXXC motifs coordinated in a finger-type arrangement^10^. The structure and length of the E7 zinc-finger is unique and not shared by any cellular protein with zinc-finger DNA-binding domains^10,18^. To date, structural characterisations by X-ray crystallography of CR3 have only been achieved using short constructs of the HPV-1a^19^ and HPV-45 variants^10^. In both structures, CR3 adopts a folded three-dimensional conformation with a relative arrangement of hydrophobic surfaces that facilitates its dimerization at native conditions.

To contribute to the understanding of the structural mechanisms associated with HPV-related diseases, high-resolution information of the full-length protein expressed by the most high-risk variant are necessary but the entire protein failed to produce X-ray crystals due to its heterogeneous structure. The use of NMR spectroscopy, instead, offers particularly favourable opportunities for the characterisation of proteins that populate simultaneously both ordered and disordered states^20–23^. Recently, an NMR characterisation of E7 has been reported with a nearly complete sequence-specific assignment of ^15^N, ^1^H^N^, ^13^C^α^, ^13^C′ NMR chemical shifts^12,24^. The NMR chemical shifts together with ^15^N relaxation measurements confirmed a heterogeneous structural and dynamical behaviour, with a highly flexible E7N and a more structured E7C. Despite the latter domain appeared to be folded, the low concentration of the sample required to prevent protein aggregation^12,24^ and the heterogeneous intensity of the NMR lines prevented the obtainment of its solution structure. Indeed, the analysis of NOESY experiments revealed only intra-residue and sequential correlations, insufficient to validate a structural model.

In this study we provide a detailed structural description of the full-length E7 in terms of a structural ensemble at nearly atomic resolution. The structural ensemble was determined using all-atom replica averaged metadynamics (RAM) simulations restrained with experimental measurements, a method that combines the sampling efficiency of metadynamics with the on-the-fly modification of the force field with experimental measurements^25^. For this purpose, we have additionally measured HN residual dipolar couplings (RDCs) and complemented them with the previously assigned ^15^N, ^1^H^N^, ^13^C^α^, ^13^C′ and ^13^C^β^ NMR chemical shifts (BMRB entries 19442 and 26069) as experimental restraints. NMR chemical shifts and RDCs are simple population-weighted observables averaged over all conformers that interconvert on timescales faster than the millisecond. As such, they can provide information about the dynamics of structural fluctuations of proteins enabling the determination of the structural ensembles that they populate^20^. The combined use of these experimental data enabled us to obtain a converged sampling in the metadynamics simulation and to characterise major structural features of the high-risk HPV-16 E7 in the context of the full-length protein.

## Results

### The free energy landscape of E7

In addition to previously reported assignments of the backbone ^15^N, ^1^H^N^, ^13^C^α^, ^13^C′ and ^13^C^β^ NMR chemical shifts^12,24^, here we measured RDCs for the backbone amide groups (HN) of E7 under conditions of fractional protein alignment (see Materials and Methods). The HN signals of the residues of E7C in the full-length construct were too weak and broad to provide measurable RDCs. Instead, we were able to determine 31 HN RDC values for the residues of E7N, that were subsequently used in the structure calculations for the full length E7 (Table S1).

To extract information about the structure and dynamics of E7 provided by the measured chemical shifts and RDCs, we incorporated them as replica-averaged structural restraints in molecular dynamics simulations, using the replica-averaged metadynamics (RAM) method^25,26^ (see Materials and Methods). In this approach, the sampling of the conformational space is carried out in an enhanced manner using the metadynamics method, and the force field is modified on-the-fly using experimental data as replica-averaged structural restraints^25,26^; as a result an ensemble of conformations is generated that is consistent with the maximum entropy principle^27–30^. In this view, the generated ensemble is the most probable one given both the force field and the experimental data used. The RAM method has been previously shown to accurately characterise the free energy landscape of folded proteins^31,32^ and, what is more challenging, the free energy landscape of denatured states^26,33,34^ and intrinsically disordered proteins^35–37^.

**Figure 1:**
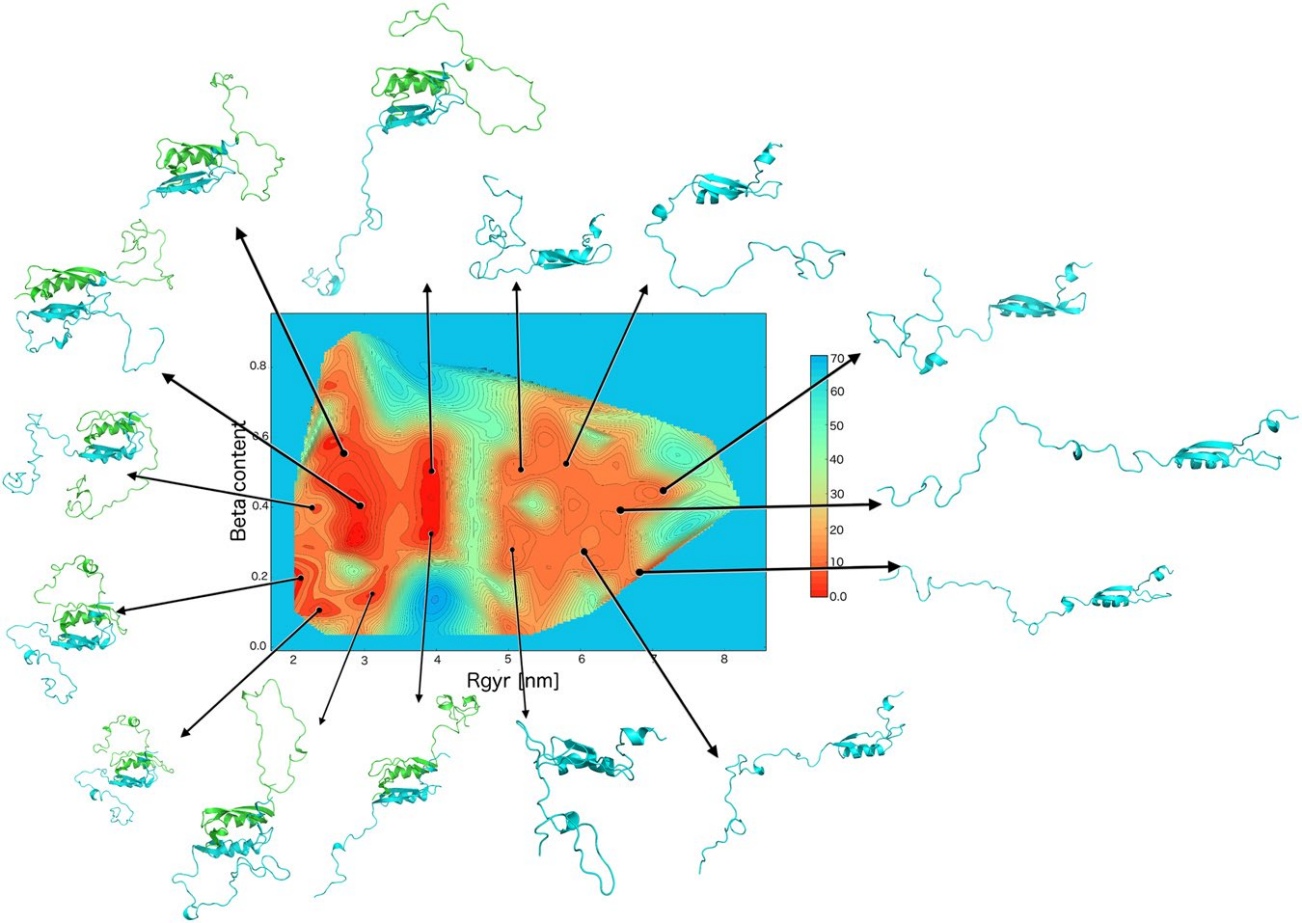
Free energy landscape of HPV-16 E7 representing the monomer-dimer equilibrium. The free energy landscape from the RAM ensemble is plotted as a function of the radius of gyration of the dimeric system (x-axis) and the number of 3-residue segments with antiparallel β-strand content in the disordered E7N domain of monomer 1 (y-axis). Representative conformations of E7 are shown in light blue; in the dimer state, the second monomer is shown in green. The antiparallel β-strand content in the disordered E7N domain is generally low and mainly comprises of short β-bridges of less than 3 residues in length. Radius of gyration values above 4.3 nm stem from the dissociation of the dimer into two monomers and represent various conformations of the isolated monomers. Representative states of both the monomer and the dimer are associated with different regions of the free energy landscape.

Application of the RAM method to full-length E7 provides the description of the structure and dynamics at a fraction of computational cost needed for the standard molecular dynamics simulations to reach convergence^25^. After a total simulation time of 2.4 μs, we obtained a converged sampling resulting in a free energy landscape within a statistical uncertainty of less than 2 kJ/mol (Fig. 1). The structures in the final ensemble cover a broad range of values of the collective variables (CVs, see Materials and Methods) used in the simulations (Figure S1): the number of transiently formed antiparallel β-strand segments in E7N of both monomers, between 0 and 4 three-residue segments; the sum of backbone dihedral angles in E7N of both monomers, between 60 rad and 120 rad, and the radius of gyration of the system, between 2.0 nm and 7.5 nm, with values above 4.3 nm corresponding to the dissociation of the dimer into two monomers. The effects of the chemical shift and RDC restraints on the underlined Amber03w force field^38^ were very small during the simulations, with an average energy of 190 kJ/mol per replica, a value about 4 orders of magnitude smaller than the average total energy of 1.43 × 10^6^ kJ/mol per replica. These results illustrate how the subtle but highly quantitative corrections to the force field introduced by the replica-averaged experimental restraints induce significant changes in the structural ensemble because of the weakness and transient nature of the interactions between different regions of disordered proteins. The changes to the E7 ensemble are illustrated by comparing the RAM free energy landscape (Fig. 1) with the free energy landscape obtained using the same procedure, but without experimental restraints (Figure S2).

### The E7 ensemble reveals an equilibrium between monomers and dimers

The RAM ensemble of E7, as described above, can be visualised by its free energy landscape plotted as a function of the radius of gyration and the number of transiently formed β-strand segments in the E7N domain (Fig. 1). A clear feature that emerges from this free energy landscape is that E7 is characterised by two well-separated basins, corresponding to a monomeric state and a dimeric state. The most populated basin corresponds to dimeric conformations with two subunits interacting mainly through the solvent-exposed hydrophobic and polar residues in E7C, homologous to E7 proteins from HPV-1a^19^ and HPV-45^10^ variants. The low populated basin corresponds to two dissociated monomers. The dimeric basin appears in the free energy landscape with a free energy of 10 ± 2 kJ/mol lower than the basin with two dissociated monomers. The two basins have therefore statistical weights of 98% and 2%, respectively, derived from the Boltzmann distribution at room temperature. The presence of a monomeric state is unlikely to be induced by the force field used in the simulations, as the population of the monomeric state is much lower in the unrestrained ensemble, with the dimeric state having a free energy by 14 ± 2 kJ/mol lower than the monomeric state (Figure S2). Another characteristic that can be extracted from the free energy landscape of E7 (Fig. 1) is that within each of the two basins there are several local minima, which is a feature typical of free energy landscapes of intrinsically disorder proteins^26,39^. This conclusion is confirmed by the broad distribution of the root mean square distances between Cα atoms (Cα-rmsd) calculated among all pairs of E7 monomers in both monomeric and dimeric states (15 ± 3 Å).

### Characterisation of the structure and dynamics of the ordered E7C domain

**Figure 2:**
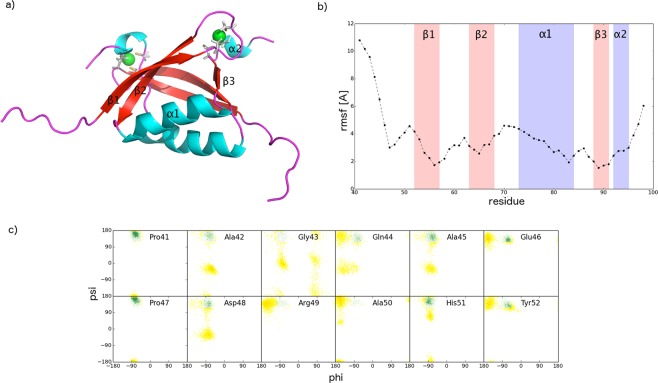
Analysis of the structural properties of the ordered E7C domain. (**a**) Ribbon representation of the secondary structure present in E7C taken from a snapshot of the RAM ensemble; (**b**) Rmsf (Å) calculated by considering only the E7C domain in the RAM ensemble; α-helices are shadowed in purple, β-sheets in pink and the ‘linker’ region (residues 41–52) that connects the disordered domain E7N with the globular domain E7C in light grey. (**c**) Ramachandran maps of residues 41–52 in the linker region. The PPII region of the Ramachandran map^41^ is coloured in green.

Close inspection of the E7C domain in the calculated structural ensemble reveals a globular domain with a β1β2α1β3α2 secondary structure connectivity (Fig. 2a), homologous to structures of E7C from the HPV-1a^19^ and HPV-45^10^ variants. A main antiparallel β-sheet is formed by the β1 (residues 52–57) and β2 (residues 63–68) strands, which pack against the main α1 helix (residues 73–84), and the short β3 strand (88–91) that extends into right-handed α2 helix (residues 92–95) (Fig. 2a). The folded structure is connected to CR2 with an 11-residue long coiled structure (residues 41–51), which we refer to as the ‘linker’ region. The CXXC motifs coordinating the Zn^2+^ ions are located in the turn connecting β1 and β2 strands and in the short C-terminal α2 helix (Fig. 2a). Zn^2+^ is tetrahedrally coordinated to the Cys residues (Cys59, Cys61, Cys91 and Cys94) and this geometry is maintained in all structures.

**Figure 3:**
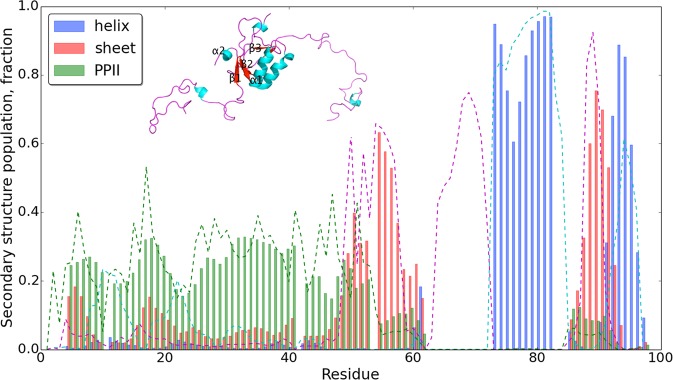
Agreement between chemical shift- and ensemble-derived secondary structure populations of E7. Secondary structure populations of E7 derived from backbone ^15^N, ^13^C′, ^13^C^α^, ^13^C^β^, ^1^H^N^ chemical shifts using the δ2D method (bars). For comparison, secondary structure populations are also obtained using an alternative method, by back-calculating them from the RAM ensemble; they are depicted by the dotted blue (α-helix), red (β-sheet) and green (PPII) lines (the remaining population, which is random coil, is not depicted here for clarity). The stretches with missing bars are due to missing chemical shift assignments in the NMR spectra and mainly concern the folded part of the E7C domain. The average structure of the E7C domain calculated from the RAM ensemble is depicted in the inset and shows partial population of β strands, in agreement with the secondary structural population.

To characterise in more detail the conformational heterogeneity of the E7C domain, we calculated the root mean square fluctuations (rmsf) of the backbone positions in the RAM ensemble by only considering the E7C domain (Fig. 2b). Rmsf values were averaged for each residue and showed, as expected, the largest values for the 11-residue linker region. This linker in the RAM ensemble populates random coil and polyproline II (PPII) conformations (total populations of 79% and 21%, respectively), which is matching closely the values calculated from the experimental chemical shifts using the δ2D method^40^ (Fig. 3). The presence of PPII conformations in this linker is confirmed in the Ramachandran map extracted from the RAM ensemble (Fig. 2c), where several residues including Ala42, Ala45, Glu46, Asp48, His51 and Tyr52 (beside Pro41 and Pro47) have a certain population of ψ/φ angles in the PPII-defined region of the conformational space^41^.

The rmsf profile for the folded part of the E7C domain shows larger values than those typically observed for folded domains^42^ (Fig. 2b). This feature is also reflected in the secondary structural population of the E7C domain (Fig. 3). In particular, the antiparallel β-sheet can be considered as being marginally stable with its constituent β strands showing significant populations of random coil conformations (β1 up to 60%, β2 up to 35% and β3 up to 20%). This finding was expected from the secondary structural populations extracted from the experimental chemical shifts^12,24^ and further confirmed by the average structure calculated from the RAM ensemble (Fig. 3, inset). The dynamic nature of the E7C domain was previously inferred based on the relatively high ^15^N transverse relaxation rates (R_2_) measured for this domain^12^.

**Figure 4:**
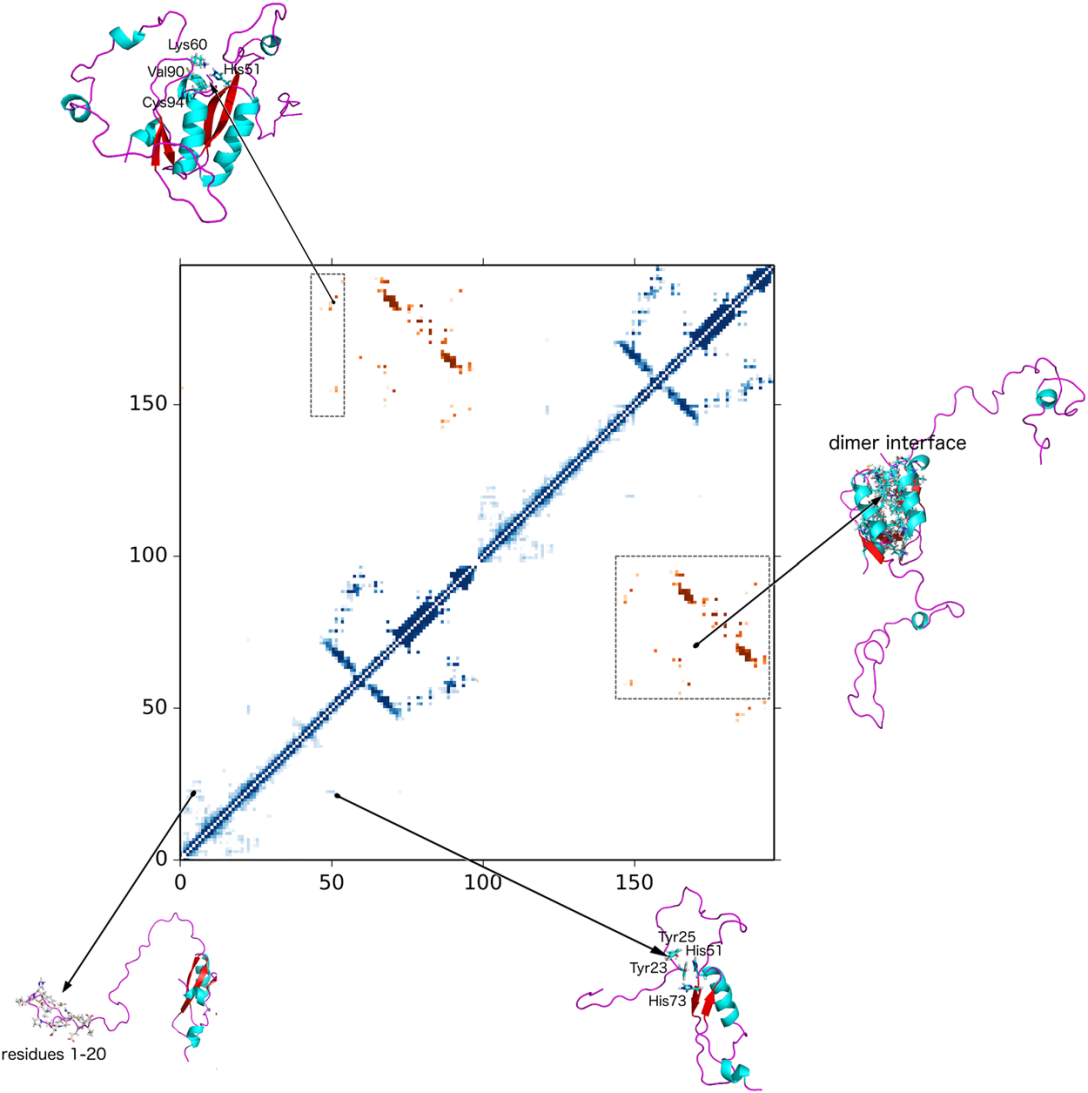
Contact map representing the monomer-dimer equilibrium of E7. Inter- and intra-molecular contacts in the RAM ensemble are depicted by orange and blue colours, respectively. Residues 1–98 (monomer one) and residues 99–196 (monomer two) are depicted along x and y axes. The E7N domain in the RAM ensemble is predominantly extended and involved in transient intra-molecular contacts between: (1) residues in the N-terminal end (lower left), and (2) Tyr23/Tyr25 and His51/His73 (lower right). Inter-molecular contacts are present at the dimer interface and these contacts superimpose well with the contacts present in the structures of low-risk HPV-1a and HPV-45 variants (Figure S3). Interestingly, residue H51 is placed at the exit of the folded part of E7C and makes both inter- and intra-molecular contacts (Fig. 5). Intermolecular contacts are made with the small helix α2 (Val90, Cys94) and β1-β2 loop Lys60) of E7C (top). This same residue makes intramolecular contacts with Tyr23 (lower right).

**Figure 5:**
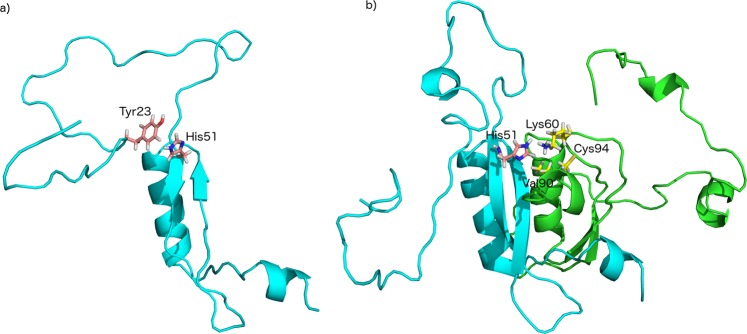
The linker region in E7 can form both intra- and inter-molecular contacts. We illustrate this conformational heterogeneity by presenting two structures in which His51 forms intra-molecular contacts with Tyr23 in the monomer (left structure) and inter-molecular contacts with Lys60, Val90 and Cys94 in the dimer (right structure).

To understand how the dimer interface in HPV-16 E7 relates to the one present in the structures of HPV-1a^19^ and HPV-45^10^, we analysed the contact map of the RAM ensemble. An analysis of this contact map (Figs 4, 5 and S3) reveals that the dimer interface is conserved across the three proteins despite the relatively low sequence identity of the three domains (sequence identity of 38% and 42% with HPV-1a and HPV-45 E7C domains, respectively). The contact residues in the RAM ensemble involve hydrophobic residues (Lys60, Leu67, Val69, Ile76, Leu79, Leu82, Gly85, Leu87, Gly88, Ile89, Val90 and Pro92), polar residues (His51, Cys59, Cys68, Gln70, Ser71, Thr72, Thr78, Thr86, Cys91, Cys94, Ser95 and Asp75).

### Structure and dynamics of the disordered E7N domain

**Figure 6:**
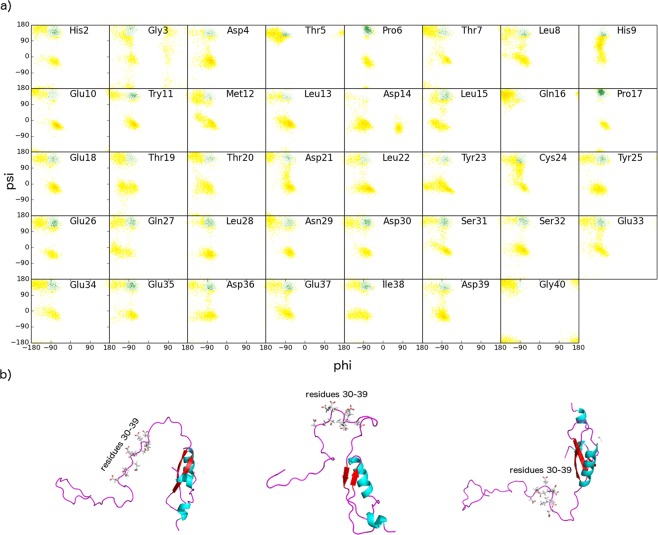
Polyproline II conformations are frequently populated by residues 1–40 in the disordered E7N domain. (**a**) The PPII region of the Ramachandran map^41^ is coloured in green; interestingly, almost all residues in the disordered E7N domain populate transiently this region. (**b**) Examples of the E7 structures populated by the extended acidic-rich region (residues 30–39). In all conformations of the RAM ensemble, the acidic-rich region is in extended conformation with two serine residues (Ser31 and Ser32) fully accessible to possible post-translational modification.

Close inspection of the structures of the E7N domain in the RAM ensemble reveals the presence of high flexibility and substantial disorder. Most of the residues of this domain form a random coil structure with a total population of 66 ± 5% (Fig. 3). In addition to the coiled conformation, the residues in the E7N domain show a propensity to form PPII conformations, which account for 26 ± 5% of the total secondary structure populations (Fig. 3). The presence of PPII regions in the RAM ensemble is also confirmed in the Ramachandran maps of residues 1–40 (Fig. 6a), in which the PPII region is occupied, at least transiently, by the majority of residues. The PPII motif is especially present in the region with unusual amount of acidic side chains that stretches over residues 30 to 39 (total population of 39 ± 5%, Fig. 3), in agreement with CD measurements^9^. α-helices in the RAM ensemble are mainly present as 3_10_ helices of short length at the N-terminal of the E7N domain and their total population is below the standard error of δ2D (below 5%). The total population of β-sheet is little above the standard error of δ2D (population of 7 ± 5%), and is mainly located at the N-terminal end of the structure.

In order to understand the level of compaction of E7N, we then analysed the tertiary contacts present in the contact map of the RAM ensemble (Figs 4 and 5). From this analysis it is evident that the E7N domain mainly exists in extended conformations. Transient contacts are only formed between N-terminal residues and the rest of E7N and between residues Tyr23/Tyr25 in E7N and His51/His73 in E7C. The former contacts are formed within the flexible N-terminal end of E7N that tends to fold back and form transient contact with the rest of E7N (Fig. 4, lower left). The latter contacts are formed between the E7N and E7C domains and stem from ring stacking between Tyr23, Tyr25, His51 and His73 (Fig. 4). Interestingly, it was previously shown that residues in this region (residues 24–26) have elevated ^15^N R_2_ rates in comparison to the rest of the E7N domain^12^ and this could be a consequence of the exchange process on the NMR time scale. These tertiary contacts are present in 27% of structures in the ensemble, and hence make the LXCXE motif (LYCYE in HPV-16, residues 22–25) not always fully accessible to bind pRb. When it comes to secondary structure, the LXCXE motif is in the extended conformation with a clear propensity for random coil (population of 74 ± 5%) and PPII (population of 21 ± 5%).

Unlike the LXCXE motif, the CKII phosphorylation site composed of S31-S32 is in the extended conformation in all structures of the RAM ensemble. These two residues are part of the acidic-rich stretch (residues 30–39, Fig. 6b), whose extended conformation is stabilised by electrostatic repulsion of the negative side chains. When we calculate electrostatic potential^43,44^ of the whole HPV-16 E7 monomer, some interesting features emerge (Figure S4). The most obvious feature is the overall negative potential coming from HPV-16 E7 with smaller regions of positive potential. This large negative potential likely plays a significant role in the conformation of HPV-16 E7; it keeps E7N domain extended and not bound to the folded E7C. Qualitative examination of the electrostatic potential shows largest values for residues 30–39 (E7N), 11-residue linker of the E7C domain and the surface of the folded E7C domain on the opposite side of the dimer interface. The only regions with mixed positive and negative potentials are N-terminal of the E7N domain and the dimer interface of the E7C domain.

### Validation of the RAM ensemble

The E7 ensemble was first validated by back-calculating NMR chemical shifts using the Sparta + predictor, which was not used during the RAM simulation^45,46^, and by comparing the obtained values to the experimentally measured chemical shifts. The RMSDs for each nucleus type were: 1.16 ppm (^15^N), 0.60 ppm (^13^Cα), 0.58 ppm (^1^Cβ), 0.49 ppm (^13^C′), and 0.24 ppm (^1^H_N_) (Figure S5). These values are within the errors of the Sparta + predictor^45^. When we generated an ensemble of E7 using the same RAM approach with the same set-up and force field but without the experimental restraints, the RMSDs between back-calculated and experimental chemical shifts increased to about 40%: 1.58 ppm (^15^N), 0.83 (^13^Cα), 0.78 (^1^Cβ), 0.70 (^13^C′), and 0.38 (^1^H_N_).

Secondary structure populations in the RAM ensemble were validated using chemical shift-based secondary structure predictor δ2D that calculates secondary structural population from the measured chemical shifts (Fig. 3, bars). These values were compared to the secondary structure population calculated from the RAM ensemble using DSSP^47^ and a PPII definition reported recently^41^ (Fig. 3, dashed line). We found high coefficient of correlations for the α-helical content (0.93, p < 0.001), for the β-sheet content (0.77, p < 0.001) and for the PPII content (0.81, p < 0.001), which confirms the convergence of the RAM simulations. Overall, the differences in secondary structural populations (Fig. 3) are within the uncertainties of the methods used to calculate them (standard errors of both δ2D and DSSP are about 5%).

The E7 ensemble was then cross-validated against the experimentally measured HN RDCs used as restraints in the simulation. In this procedure, different sets of experimentally measured RDCs were used for tensor calculation from the ensemble and for back calculation of RDCs. The level of agreement was very high, with an average value of Q factor of 0.27 (Figure S5). This agreement is significantly better than that for the unrestrained ensemble (Q factor of 0.95).

The ensemble was verified also against the experimentally determined Stokes radius for the E7N (1–40) peptide^9^. When the radius of gyration of residues 1–40 was back-calculated from the RAM ensemble, it produced an overall average value of R_g_ = 1.70 ± 0.34 nm. This value corresponds to a Stokes radius of 2.04 ± 0.40 nm, which is consistent with the measured experimental values of 2.09 nm and 2.13 nm^9^.

**Figure 7:**
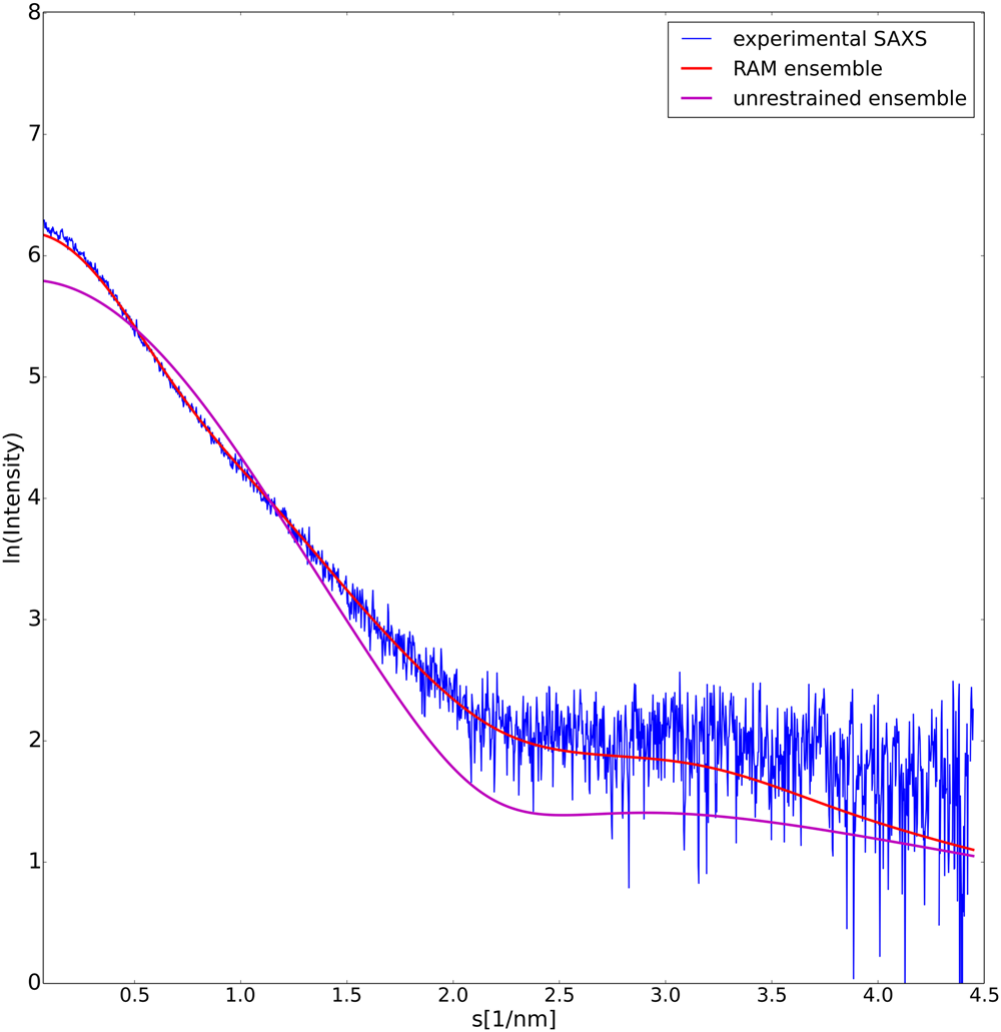
Validation of the RAM ensemble using SAXS measurements. Comparison between experimental (blue line) and back-calculated SAXS profiles from: (**a**) RAM ensemble (red line), (**b**) unrestrained ensemble (magenta line). The better agreement for the RAM ensemble, in particular for low values of s (see also Figure S6), indicates that the restraints greatly improved description of the overall shape of the molecule.

Finally, the ensemble was validated by recording small-angle-X-ray scattering (SAXS) data  on the entire E7 protein at increasing protein concentration values. According to the Guinier approximation^48^ the scattering intensity plotted as ln I(s) versus the squared momentum transfer is a linear function, were R_g_ is the slope of the linear fit. Our data on full-length E7 (Figure S6), provide a R_g_ = 3.34 ± 0.05 nm, a value that matches the average value of the dimeric state provided by the energy landscape, R_g_ = 3.16 nm. Furthermore, the SAXS profile back-calculated and averaged over from the RAM ensemble using Crysol^49^ was highly consistent with the experimental profile (χ^2^ = 0.83) (Fig. 7). The average SAXS profile back-calculated from the unrestrained ensemble using Crysol showed no compatibility with the experiment (χ^2^ = 3.56) (Fig. 7).

## Discussion

Although conformationally heterogeneous states of proteins are involved in a wide range of biological processes, a detailed knowledge of their structure and dynamics is still very challenging to obtain. This is also the case for the oncogenic protein E7 of HPV-16, which is formed by the mainly globular E7C domain and the disordered E7N domain. Indeed, it has been shown crucial to obtain atomic information of E7 in its full form, as different components of the protein appear to have complementary roles when interacting with cellular partners and shorter constructs display lower affinities^4,16,50^. Therefore, the determination of structure and dynamics of the full-length E7 obtained in this study, including the presence of equilibrium between a monomeric state and a dimeric state, offers an opportunity to uncover the presence and functional relevance of its conformationally heterogeneous states.

We have obtained here a structural ensemble representing the monomer/dimer equilibrium of full-length E7 using NMR chemical shifts complemented with HN RDCs measured for the purpose of this study. The experimental measurements were incorporated as replica-averaged structural restraints in molecular dynamics simulations, using the RAM method, and produced an ensemble of conformations according to the maximum entropy principle^27,28^. The free energy landscape of E7 shows that the dimer conformation is dominant, and that it is stabilised by 10 ± 2 kJ/mol in comparison to the monomeric conformation. The results are in agreement with structural evidence available on the construct of E7C from different viral types that showed the presence of the dimeric state at neutral pH^10,19^. Moreover, the analysis of ^15^N relaxation rates of E7 from HPV-16 in its full form also suggests that E7C is affected by conformational exchange^12,24^. The existence of the predominant dimeric form in solution was further confirmed by SAXS data on the full-length protein.

Although there is limited atomic resolution information for the E7N domain for any HPV virus variant, this domain has been broadly characterised as disordered^12,13,51^. Therefore, one of our aims here was to give a more detailed picture of the E7N domain structure in order to better understand its high promiscuity in the cell^13^. The structural ensemble that we have reported here confirms the overall high conformational heterogeneity of this domain, which can adopt both compact and extended conformations and is characterised with high percentage of random coil population. However, the structural ensemble also demonstrates that the E7N domain does not behave as a random coil with no preferred backbone conformation. Rather, this domain tends to adopt a PPII conformation along the whole sequence with a total population of 26%. Interestingly, 23 consecutive residues stretching between P17 and I39 have a significant population of φ/ψ angles characteristic of PPII in the Ramachandran plot. This extended conformation is caused by highly negative electrostatic potential surface of the E7 monomer with a net charge of −16.

Apart to the stability contribution, the extended conformations might have a specific functional significance, as they can promote binding to many different interaction partners by increasing accessibility of its interaction motifs. Indeed, the acidic-rich stretch of residues 30–39 have the highest tendency to populate PPII (39 ± 5%), which is stabilized by electrostatic repulsion of its negatively charged side chains. Residues S31 and S32 are positioned in this acidic-rich region, which make them accessible to phosphorylation by CKII that in turn further increases the PPII content of the E7N domain^9^. Moreover, keeping E7N and E7C apart might be an important feature to render them independent one from the other and fully accessible, but still have complementary roles when interacting with cellular partners positioned next to each other.

## Conclusions

The atomic resolution structural ensemble of E7 from HPV-16 reported here reveals very heterogeneous structural and dynamical properties that go beyond the current schematic view of a disordered E7N domain and an ordered E7C domain, and involve an equilibrium between a low populated monomeric state and a high populated dimeric state. The availability of this structural ensemble opens the possibility of atomic resolution characterisations of post-translational modifications and interactions with many partners known to bind different motifs in E7 of HPV-16. As applications of the RAM simulations restrained with experimental measurements, as well as other complementary methods for characterising protein structural ensembles^23,39^, continues to grow so too will our understanding of how protein dynamics control function. These developments will offer an increasingly detailed understanding of structure-dynamics-function relationship in HPV-16 E7, and of intrinsically disordered proteins more in general.

## Materials and Methods

### NMR measurements of HN RDCs of E7N

^15^N labelled E7 from HPV-16 was expressed and purified as previously reported^12^. A sample of 150 μM ^15^N labelled E7 in 50 mM HEPES, 1 mM DTT, 50 mM KCl at pH 7.5 was used for the measurement of backbone HN RDCs.

^1^H-^15^N backbone splittings were measured through the IPAP approach^52^. The in-phase and antiphase components of the ^15^N signal in indirect dimension of 2D ^1^H-^15^N correlation spectra were acquired and stored separately. By appropriate linear combinations the two doublet components were separated and used to determine the splitting in the ^15^N indirect dimension. For each cross peak, traces in indirect dimension were extracted. The ^1^H-^15^N splittings were determined by shifting one trace respect to the other. The 2D ^1^H-^15^N NMR experiments were acquired with 4 scans per increment (32 dummy scans), with a relaxation delay of 1.3 s and with 3072 × 2048 data points and 16.2 × 36 ppm in the ^1^H direct and ^15^N indirect dimensions, respectively. The experiments were acquired on the ^15^N labelled E7 sample before and after addition of the orienting medium. The difference between the ^1^H-^15^N splittings provides the HN RDCs, with an estimated error of ±0.1 Hz^52^.

A mixture of C5E8 and octanol, which forms a dilute liquid crystalline phase, was used as orienting medium^53^. In particular, 16 μl of C_5_E_8_ and 7 μl of octanol was added to a 550 μl E7 sample (3%, R = 0.87, ^2^H splitting of 15 Hz). RDCs values could only be determined for residues belonging to the E7 N-terminal part (31 residues, Table S1) as signals for E7C were generally weak and broad, and in part not assigned.

### Molecular dynamics simulations

Molecular dynamics simulations of E7 were performed using the Amber03w force field^54^ and TIP4P/2005 water model^55^, which are particularly suitable for studying unfolded states and intrinsically disordered proteins over a wide range of thermodynamic conditions^38^. All simulations were run using GROMACS 4.5^56^ modified with PLUMED2^57^.

A starting model for HPV-16 E7 dimer was generated using homology modelling from the amino acid sequence (UniProt code P03129) and the NMR solution structure of HPV-45 E7 dimer (PDB ID: 2EWL, 65% sequence identity) used as a template for E7C. The homology modelling was performed using Rosetta software^58^ implemented on the Robetta server. The starting structure was solvated with 34,682 water molecules and neutralized with 32 Na^+^ ions in a water box of 1070 nm^3^ of volume. A high-temperature (450 K) 30 ns preliminary simulation was used to select six starting conformations for each of six replicas (see below). Each conformation was then subsequently relaxed at 300 K for 10 ns. A time step of 2 fs was used together with LINCS constraints for all simulations^59^. Van der Waals interactions were implemented with a cut-off at 0.9 nm, and long-range electrostatic effects were treated with the particle mesh Ewald method^60^.

The RAM simulations were carried out by combining two advanced sampling methods, replica exchange^61^ and metadynamics^62^. First, replica exchange is particularly effective in overcoming the multiple minima problem on a rugged free energy surface through the exchange of conformations between multiple replicas. Second, metadynamics efficiently computes free energies and explores the reaction pathways in the space of specific functions of atomic coordinates, called collective variables (CVs)^62^. After performing initial tests, we carried out RAM simulations using six replicas (five replicas biased by five CVs and one unbiased replica) that showed sufficient to recover E7 dynamics with high efficiency^25–27,63^. The following five CVs have been employed: the number of 3-residue segments with antiparallel β-strand content of E7N monomer 1 (i) and monomer 2 (ii), the sum of backbone dihedral angles of E7N monomer 1 (iii) and monomer 2 (iv), and the radius of gyration of the whole protein E7 (v). Using the RAM scheme, six replicas were simulated in parallel at 300 K with experimental restraints applied on the average value of back-calculated ^15^N, ^1^H^N^, ^13^C^α^, ^13^C′ and ^13^C^β^ NMR chemical shifts and ^1^H^N^-^15^N RDCs using a previously detailed procedure^32,63^. All replicas were simulated in the canonical ensemble at constant volume and by thermosetting the system with the velocity rescaling thermostat^64^. Each replica evolved for 400 ns (2.4 μs of simulation time in total), with exchange trials every 50 ps.

The convergence of the sampling was assessed by monitoring the differences of the free energies at increasing simulation length during the simulations. After the first 350 ns per replica, the free energy landscapes were stable within 2 kJ/mol, suggesting that all the relevant minima in the landscape have been found (Figure S1). The free energy landscape of the full length E7 was reconstructed using a standard weighted histogram analysis^65^ (Fig. 1) as a function of two CVs. The final ensemble consists of 2450 conformations of the full length E7.

### SAXS measurements of HPV-16 E7

The SAXS experiments were performed at the EMBL P12 beamline, DESY, Hamburg, Germany^66^, at E7 protein concentrations 1, 2 and 4 mg/ml in 50 mM HEPES, 1 mM DTT, 50 mM KCl, pH 7.5 at 298 K. The data were recorded using a Pilatus 2 M detector (DECTRIS, Switzerland) with 20 frames of 0.05 s exposure time, at a sample-detector distance 3.00 m and wavelength λ = 0.124 nm. No measurable radiation damage was detected by comparison of successive time frames. The data were processed with the ATSAS package^67^ using standard procedures, corrected for buffer contribution, and extrapolated to infinite dilution. The radius of gyration was computed using the Guinier approximation^48^:

$$I(s)\cong \,{I}_{0}\,{e}^{-\frac{{R}_{g}^{2}}{3}{s}^{2}}$$

where I(s) is the measured intensity, I_0_s is the forwarded scattering, R_g_ is the radius of gyration and s the squared momentum transfer. The momentum transfer s is defined by *s* = 4πsin*θ*/λ, where λ is the X-ray wavelength (0.124 nm) and 2*θ* is the scattering angle.

The goodness of fit to the experimental SAXS profile was determined using the discrepancy defines as χ^2^ = $\sum _{i=1}^{\nu }\frac{{({x}_{i}-{\mu }_{i})}^{2}}{{\sigma }_{i}^{2}}$, where µ_i_ and σ_i_ are mean and variance of the measured scattering intensities.

## Supplementary information

Supplementary Information

## Data Availability

All data generated or analysed during this study are included in this published article (and its Supplementary Information files).

## References

[1] zur Hausen H (2002). Papillomaviruses and cancer: from basic studies to clinical application. Nature Reviews Cancer.

[2] Walboomers JM (1999). Human papillomavirus is a necessary cause of invasive cervical cancer worldwide. The Journal of pathology.

[3] Eun-Kyoung Y, Jong-Sup P (2005). The Role of HPV E6 and E7 Oncoproteins in HPV-associated Cervical Carcinogenesis. Cancer research and treatment.

[4] Dona, M. G. In *Oncogene* Pro*te*ins: *New Research* (eds A Malloy & E Carson) 19–63 (Nova Science Publishers, 2008).

[5] Mantovani F, Banks L (2001). The Human Papillomavirus E6 protein and its contribution to malignant progression. Oncogene.

[6] Pim D, Banks L (2010). Interaction of viral oncoproteins with cellular targetmolecules: infection with high-risk vs low-risk humanpapillomaviruses. APMIS.

[7] Martinez-Zapien D (2016). Structure of the E6/E6AP/p53 complex required for HPV-mediated degradation of p53. Nature.

[8] Jansma AL (2014). The high-risk HPV16 E7 oncoprotein mediates interaction between the transcriptional coactivator CBP and the retinoblastoma protein pRb. Journal of molecular biology.

[9] García-Alai M, Alonso L, de Prat-Gay G (2007). The N-terminal module of HPV16 E7 is an intrinsically disordered domain that confers conformational and recognition plasticity to the oncoprotein. Biochemistry.

[10] Ohlenschläger O (2006). Solution structure of the partially folded high-risk human papilloma virus 45 oncoprotein E7. Oncogene.

[11] Uversky V, Roman A, Oldfield C, Dunker K (2006). Protein Intrinsic Disorder and Human Papillomaviruses:  Increased Amount of Disorder in E6 and E7 Oncoproteins from High Risk HPVs. Journal of Proteome Research.

[12] Calcada E, Felli I, Hosek T, Pierattelli R (2013). The Heterogeneous Structural Behavior of E7 from HPV16 Revealed by NMR Spectroscopy. ChemBioChem.

[13] McLaughlin-Drubin M, Münger K (2009). The human papillomavirus E7 oncoprotein. Virology.

[14] Moody CA, Laimins LA (2010). Human papillomavirus oncoproteins: pathways to transformation. Nature reviews. Cancer.

[15] Dyson N, Howley P, Munger K, Harlow E (1989). The Human Papilloma Virus-16 E7 Oncoprotein Is Able to Bind to the Retinoblastoma Gene Product. Science.

[16] Lee J-O, Russo A, Pavletich N (1998). Structure of the retinoblastoma tumour-suppressor pocket domain bound to a peptide from HPV E7. Nature.

[17] Firzlaff J, Galloway D, Eisenman R, Lüscher B (1989). The E7 protein of human papillomavirus type 16 is phosphorylated by casein kinase II. The New Biologist.

[18] Watanabe S, Sato H, Furuno A, Yoshiike K (1992). Changing the spacing between metal-binding motifs decreases stability and transforming activity of the human papillomavirus type 18 E7 oncoprotein. Virology.

[19] Liu, X., Clements, A., Zhao, K. & Marmorstein, R. Structure of the Human Papillomavirus E7 Oncoprotein and Its Mechanism for Inactivation of the Retinoblastoma Tumor Suppressor. *The Journal of Biological Chemistry* **281** (2006). 10.1074/jbc.M508455200 16249186

[20] Sormanni P (2017). Simultaneous quantification of protein order and disorder. Nature Chemical Biology.

[21] Brutscher, B. *et al*. In *Advances in Experimental Medicine and Biology* Vol. 870 (eds Isabella C. Felli & Roberta Pierattelli) 49–122 (Springer International Publishing, 2015). 10.1007/978-3-319-20164-1_3 26387100

[22] Dyson HJ, Wright PE (2005). Intrinsically unstructured proteins and their functions. Nature reviews. Molecular cell biology.

[23] Van Der Lee R (2014). Classification of intrinsically disordered regions and proteins. Chemical reviews.

[24] Nogueira MO (2017). Monitoring HPV-16 E7 phosphorylation events. Virology.

[25] Camilloni C, Cavalli A, Vendruscolo M (2013). Replica-Averaged Metadynamics. Journal of Chemical Theory and Computation.

[26] Camilloni C, Vendruscolo M (2014). Statistical mechanics of the denatured state of a protein using replica-averaged metadynamics. Journal of the American Chemical Society.

[27] Cavalli A, Camilloni C, Vendruscolo M (2013). Molecular dynamics simulations with replica-averaged structural restraints generate structural ensembles according to the maximum entropy principle. The Journal of Chemical Physics.

[28] Roux B, Weare J (2013). On the statistical equivalence of restrained-ensemble simulations with the maximum entropy method. The Journal of Chemical Physics.

[29] Pitera J, Chodera J (2012). On the Use of Experimental Observations to Bias Simulated Ensembles. Journal of Chemical Theory and Computation.

[30] Boomsma W, Ferkinghoff-Borg J, Lindorff-Larsen K (2014). Combining Experiments and Simulations Using the Maximum Entropy Principle. PLoS Comput Biol.

[31] Kukic P (2015). Structure and Dynamics of the Integrin LFA-1 I-Domain in the Inactive State Underlie its Inside-Out/Outside-In Signaling and Allosteric Mechanisms. Structure.

[32] Kukic P (2016). Structural Insights into the Calcium-Mediated Allosteric Transition in the C-Terminal Domain of Calmodulin from Nuclear Magnetic Resonance Measurements. Biochemistry.

[33] Granata, D., Camilloni, C., Vendruscolo, M. & Laio, A. Characterization of the free-energy landscapes of proteins by NMR-guided metadynamics. *Proceedings of the National Academy of Sciences USA* **110**, 6817–6822 (2013). 10.1073/pnas.1218350110 PMC3637744 23572592

[34] Kukic P (2017). Structural Characterization of the Early Events in the Nucleation–Condensation Mechanism in a Protein Folding Process. Journal of the American Chemical Society.

[35] Kitevski-LeBlanc J (2017). The RNF168 paralog RNF169 defines a new class of ubiquitylated histone reader involved in the response to DNA damage. eLife.

[36] Toto, A. *et al*. Molecular Recognition by Templated Folding of an Intrinsically Disordered Protein. *Scientific Reports*, **21994** (2016). 10.1038/srep21994 PMC4766501 26912067

[37] Yoshimura Y (2017). MOAG-4 promotes the aggregation of α-synuclein by competing with self-protective electrostatic interactions. Journal of Biologial Chemistry.

[38] Best R, Mittal J (2010). Protein Simulations with an Optimized Water Model: Cooperative Helix Formation and Temperature-Induced Unfolded State Collapse. The Journal of Physical Chemistry B.

[39] Bonomi M, Heller G, Camilloni C, Vendruscolo M (2017). Principles of protein structural ensemble determination. Current Opinion in Structural Biology.

[40] Camilloni C, De Simone A, Vranken WF, Vendruscolo M (2012). Determination of Secondary Structure Populations in Disordered States of Proteins Using Nuclear Magnetic Resonance Chemical Shifts. Biochemistry.

[41] Mansiaux Y, Joseph A, Gelly J-C, de Brevern A (2011). Assignment of PolyProline II Conformation and Analysis of Sequence – Structure Relationship. PLoS One.

[42] Vendome J (2011). Molecular design principles underlying β-strand swapping in the adhesive dimerization of cadherins. Nature Structural and Molecular Biology.

[43] Baker, N., Sept, D., Joseph, S., Holst, M. & McCammon, A. Electrostatics of nanosystems: Application to microtubules and the ribosome. *Proceedings of the National Academy of Sciences USA* **98** (2001). 10.1073/pnas.181342398 PMC56910 11517324

[44] Kukic P (2013). Protein Dielectric Constants Determined from NMR Chemical Shift Perturbations. Journal of the American Chemical Society.

[45] Shen Y, Bax A (2010). SPARTA+: a modest improvement in empirical NMR chemical shift prediction by means of an artificial neural network. Journal of Biomolecular NMR.

[46] Kohlhoff KJ, Robustelli P, Cavalli A, Salvatella X, Vendruscolo M (2009). Fast and Accurate Predictions of Protein NMR Chemical Shifts from Interatomic Distances. Journal of the American Chemical Society.

[47] Kabsch W, Sander C (1983). Dictionary of protein secondary structure: Pattern recognition of hydrogen-bonded and geometrical features. Biopolymers.

[48] Guinier, A. & Fournet, G. *Small angle scattering of X-rays*. (John Wiley and Son, 1955).

[49] Svergun D, Barberato C, Koch M (1995). CRYSOL– a Program to Evaluate X‐ray Solution Scattering of Biological Macromolecules from Atomic Coordinates. Journal of applied crystallography.

[50] Patrick D, Oliff A, Heimbrook D (1994). Identification of a novel retinoblastoma gene product binding site on human papillomavirus type 16 E7 protein. The Journal of Biological Chemistry.

[51] Lee C, Kim D-H, Lee S-H, Su J, Han K-H (2016). Structural investigation on the intrinsically disordered N-terminal region of HPV16 E7 protein. BMB reports.

[52] Ottige M, Delaglio F, Bax A (1998). Measurement of J and Dipolar Couplings from Simplified Two-Dimensional NMR Spectra. Journal of Magnetic Resonance.

[53] Rückert M, Otting G (2000). Alignment of Biological Macromolecules in Novel Nonionic Liquid Crystalline Media for NMR Experiments. Journal of the American Chemical Society.

[54] Best R, Mittal J (2010). Balance between α and β Structures in Ab Initio Protein Folding. Journal of Physical Chemistry B.

[55] Abascal J, Vega C (2005). A general purpose model for the condensed phases of water: TIP4P/2005. The Journal of Chemical Physics.

[56] Pronk, S. *et al*. GROMACS 4.5: a high-throughput and highly parallel open source molecular simulation toolkit. *Bioinformatics* **29** (2013). 10.1093/bioinformatics/btt055 PMC3605599 23407358

[57] Tribello GA, Bonomi M, Branduardi D, Camilloni C, Bussi G (2014). PLUMED 2: New feathers for an old bird. Computer Physics Communications.

[58] Rohl C, Strauss C, Misura K, Baker D (2004). Protein structure prediction using Rosetta. Methods Enzymol..

[59] Hess B, Kutzner C, van der Spoel D, Lindahl E (2008). GROMACS 4:  Algorithms for Highly Efficient, Load-Balanced, and Scalable Molecular Simulation. Journal of Chemical Theory and Computation.

[60] Darden T, York D, Pedersen L (1993). Particle mesh Ewald: An N⋅log(N) method for Ewald sums in large systems. The Journal of Chemical Physics.

[61] Sugita Y, Okamoto Y (1999). Replica-exchange molecular dynamics method for protein folding. Chemical Physics Letters.

[62] Laio, A. & Parrinello, M. Escaping free-energy minima. *Proceedings of the National Academy of Sciences USA* **99**, 12562–12566 (2002). 10.1073/pnas.202427399 PMC130499 12271136

[63] Camilloni C, Vendruscolo M (2015). A tensor-free method for the structural and dynamical refinement of proteins using residual dipolar couplings. The Journal of Physical Chemistry B.

[64] Bussi G, Donadio D, Parrinello M (2007). Canonical sampling through velocity rescaling. The Journal of Chemical Physics.

[65] Biarnes X, Pietrucci F, Marinelli F, Laio A (2012). METAGUI. A VMD interface for analyzing metadynamics and molecular dynamics simulations. Computer Physics Communications.

[66] Blanchet C (2015). Versatile sample environments and automation for biological solution X-ray scattering experiments at the P12 beamline (PETRA III, DESY). Journal of applied crystallography.

[67] Franke D (2017). ATSAS 2.8: a comprehensive data analysis suite for small-angle scattering from macromolecular solutions. Journal of applied crystallography.

