# Supplementary material for: The free energy landscape of the oncogene protein E7 of human papillomavirus type 16 reveals a complex interplay between ordered and disordered regions
Source: Sci Rep. 2019 Apr 9;9:5822. doi: 10.1038/s41598-019-41925-4 (PMC6456579; doi:10.1038/s41598-019-41925-4)
Supplement: Supplementary file 1 — Supplementary Information [file 41598_2019_41925_MOESM1_ESM.docx]

**Supplementary Information**

**The free energy landscape of the oncogene protein E7 of human papillomavirus type 16 reveals a complex interplay between ordered and disordered regions**

Predrag Kukic^1^, Giuseppe Mattia Lo Piccolo^1,2^, Marcela O. Nogueira^2^, Dmitri Svergun^3^

Michele Vendruscolo^1,*^, Isabella C. Felli^2,4^, Roberta Pierattelli^2,4^

*^1^Department of Chemistry, University of Cambridge, Cambridge CB2 1EW, UK*

*^2^Magnetic Resonance Center (CERM), University of Florence, 50019 Sesto Fiorentino, Italy*

*^3^European Molecular Biology Laboratory, EMBL Hamburg Unit, c/o DESY, Notkestrasse 85, D-22607, Hamburg, Germany*

*^4^Department of Chemistry “Ugo Schiff”, University of Florence, 50019 Sesto Fiorentino, Italy*

^*^ *mv245@cam.ac.uk*

**Table S1.** **List of HN RDCs measured for the disordered E7N region.**

| Residue # | RDC [Hz] | Residue # | RDC [Hz] | Residue # | RDC [Hz] | Residue # | RDC [Hz] |
| --- | --- | --- | --- | --- | --- | --- | --- |
| 4 | 2.15 | **20** | 1.87 | **29** | -0.04 | **37** | 3.05 |
| 5 | -0.46 | **21** | 0.91 | **30** | 2.71 | **38** | 2.84 |
| 8 | -5.26 | **22** | 0.18 | **31** | 0.19 | **39** | 3.68 |
| 11 | -3.22 | **23** | -0.19 | **32** | 0.35 | **40** | 2.66 |
| 14 | -0.87 | **25** | 1.17 | **33** | 0.80 | **42** | 2.49 |
| 16 | 0.15 | **26** | 3.25 | **34** | 3.21 | **43** | 2.53 |
| 18 | 0.86 | **27** | 1.59 | **35** | 3.67 | **44** | 3.65 |
| 19 | 3.06 | **28** | 1.46 | **36** | 2.03 |  |  |


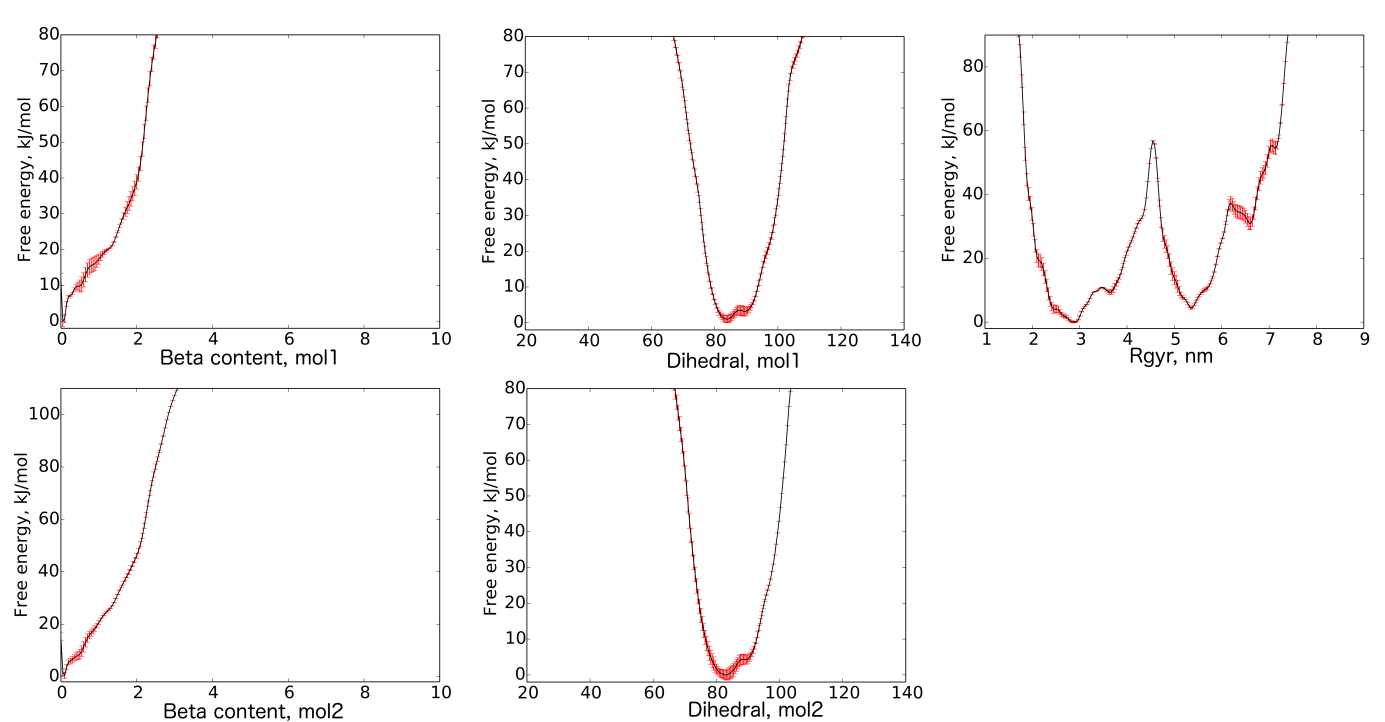


**Figure S1.** **Assessment of the convergence of the free energy calculations**. The free energy profiles (in kJ/mol) of the five collective variables (see **Materials and Methods**) are averaged over the final segment of the RAM simulations (the last 50 ns), and their standard deviations are reported as error bars (within 2.0 kJ/mol).


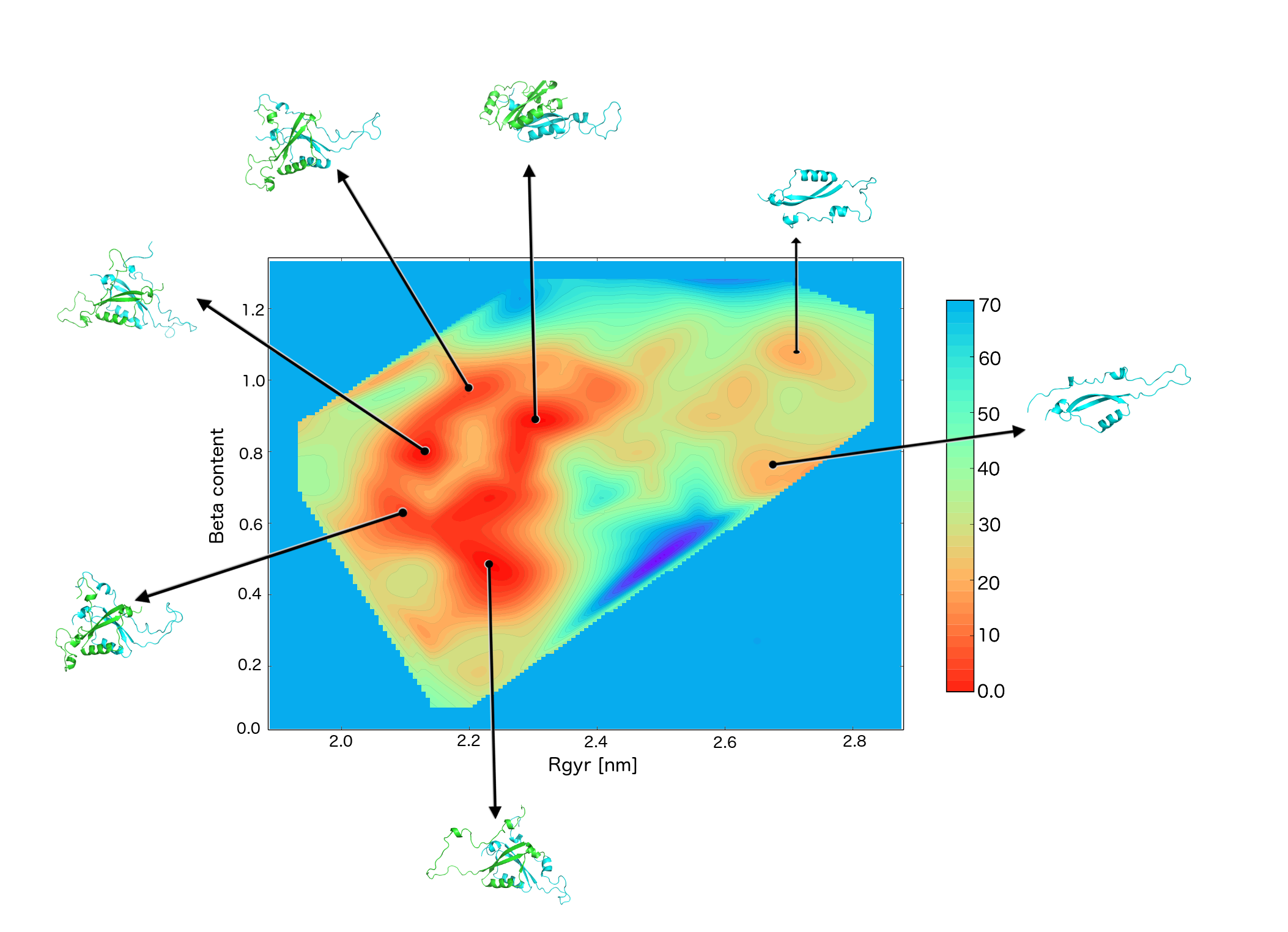


**Figure S2. Free energy landscape of HPV-16 E7 from a control unrestrained simulation.** As in **Figure 1**, the free energy landscape is plotted as a function of the radius of gyration of the whole protein (x-axis) and the number of 3-residue segments with antiparallel β-strand content in the E7N domain of monomer 1 (y-axis). The ensemble is significantly more compact than the RAM ensemble (see **Figure 1**). Radius of gyration values above 2.5 nm stem from the dissociation of the dimer into two monomers and represent various conformations of the isolated monomers. The dimeric basin appears in the free energy landscape with a free energy of 14±2 kJ/mol lower than the basin with two dissociated monomers (different from a corresponding free energy of 10±2 kJ/mol from the RAM ensemble). The unrestrained ensemble shows relatively poor agreement with the experimental chemical shifts and RDCs (see **Results**).

**
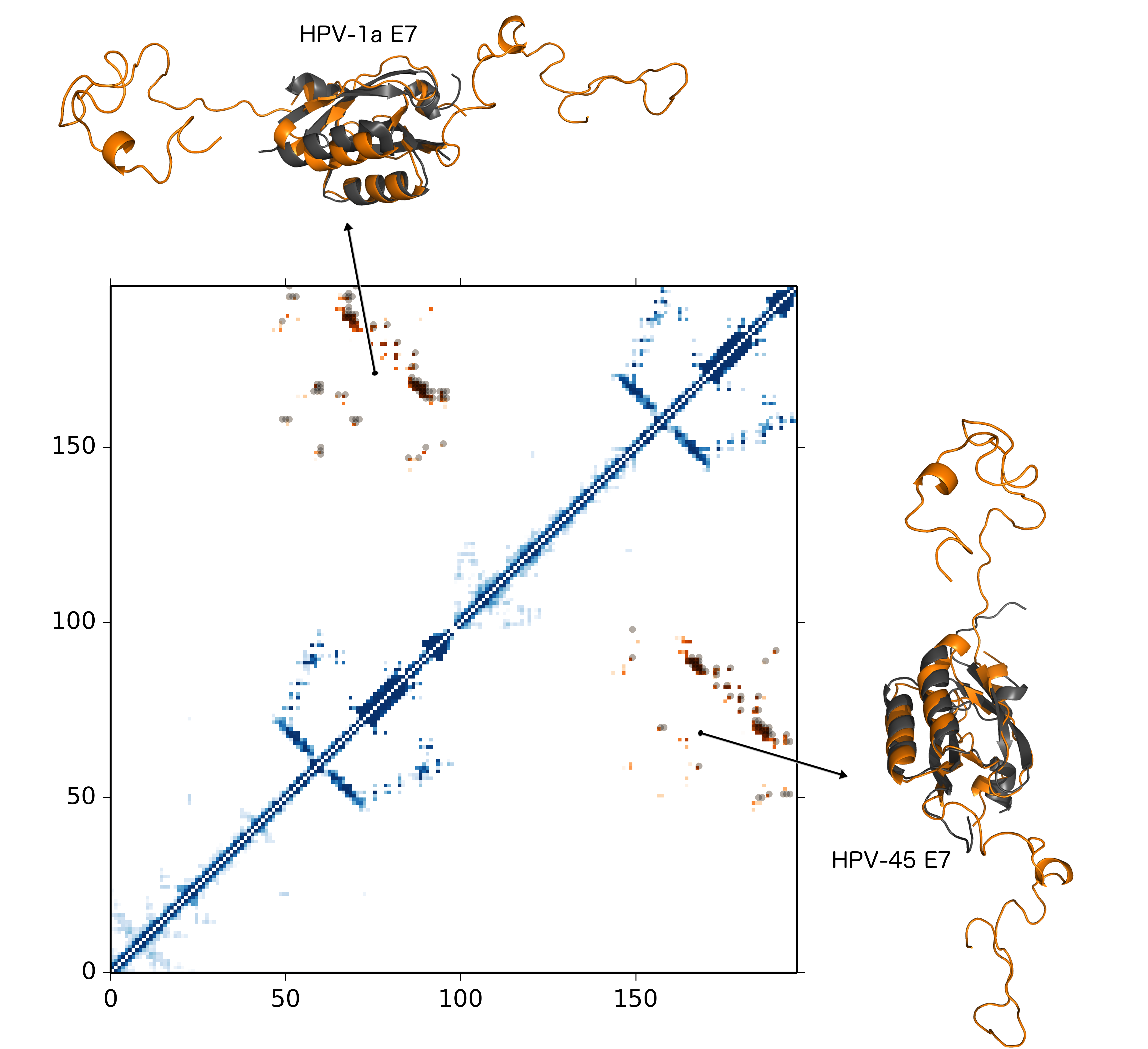
**

**Figure S3. Comparison between the contact map of E7 and the contact maps of the low-risk HPV-1a and HPV-45 variants.** The inter-molecular contacts (orange) present in the RAM E7 ensemble superimpose well with the contacts present in the structures of low-risk HPV-1a (PDB ID: 2b9d, upper grey) and HPV-45 (PDB ID: 2f8b, right grey) variants.

**Figure S4. Electrostatic properties of the HPV-16 E7 monomer**. Potential isocontours are shown at +5 kT/e (blue) and −5 kT/e (red) and obtained by solving the Poisson Boltzmann equation at 50 mM ionic strength with a solute dielectric of 4.0 and a solvent dielectric of 78.5^1^ using APBS^2^. The left view shows mixed positive and negative potential isocounters coming from the dimer interface and N-terminal, and strong negative potential in the rest of the molecule. The right view shows strong negative potential opposite the dimer interface and along the whole molecule.


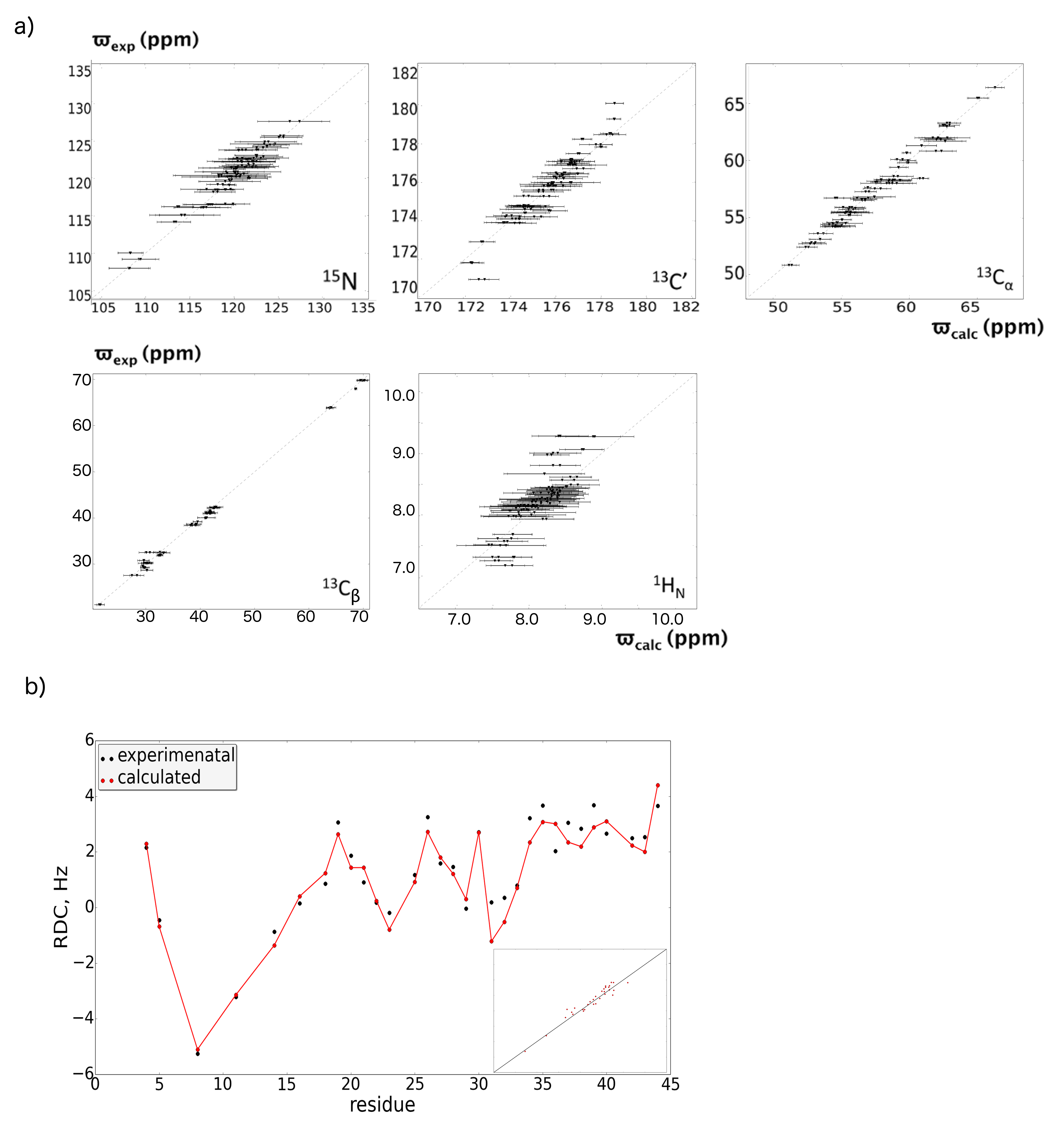


**Figure S5. Validation of the RAM ensemble.** (a) Validation of the RAM ensemble by correlating experimental (y-axis) and back-calculated (x-axis) ^15^N (RMSD of 1.16ppm), ^13^C’ (0.49 ppm), ^13^Cα (0.60 ppm), ^13^Cβ (0.58 ppm) and ^1^H_N_ (0.24 ppm) chemical shifts. (b) Validation of the RAM ensemble by comparing experimental (black) and back-calculated (red) ^1^H^N^-^15^N RDCs (Q-factor = 0.27).


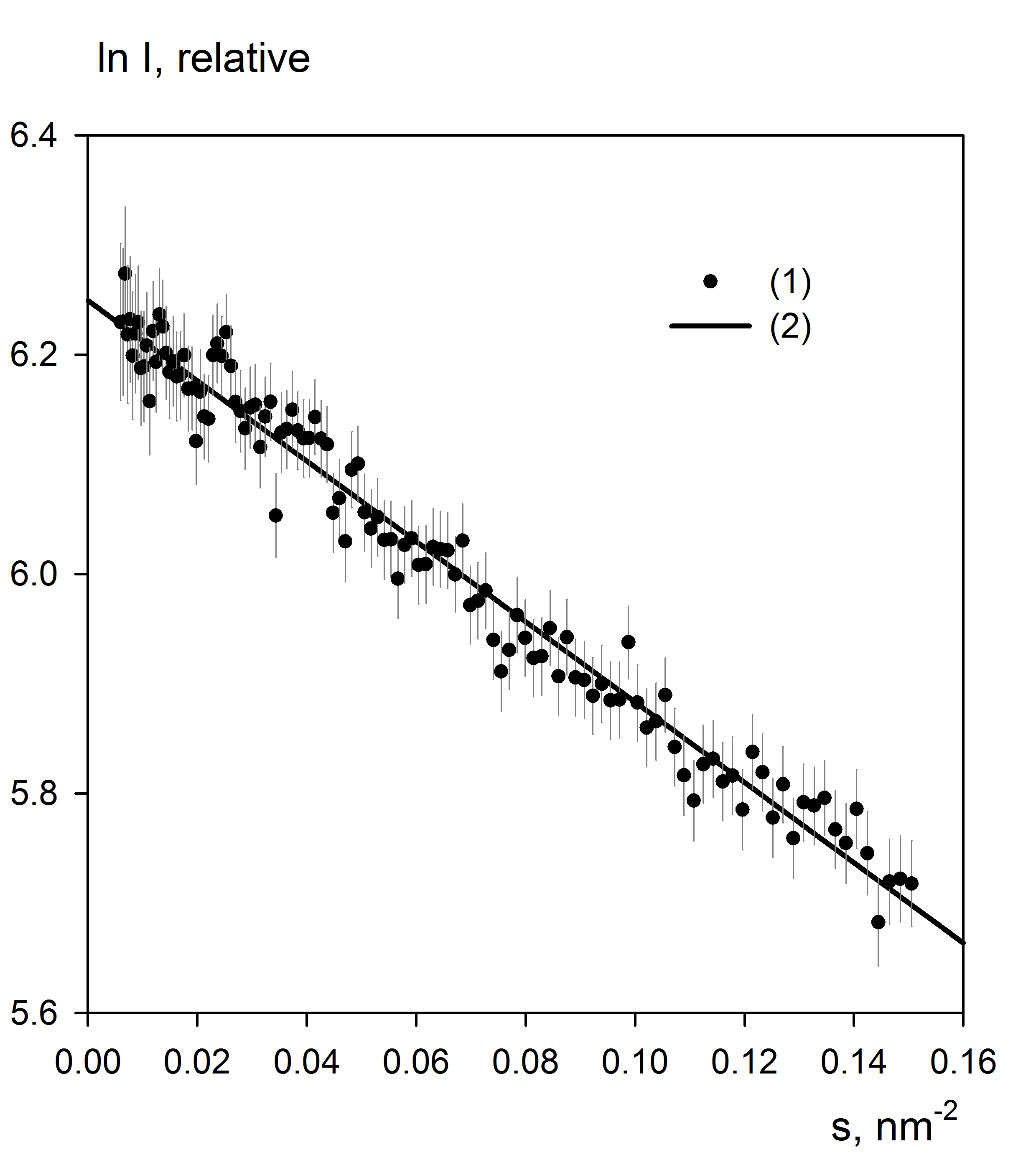


s^2^, nm^-2^

ln I, relative

**Figure S6.** The Guinier plot from the SAXS data collected on E7 in solution: (1), experimental data with error bars, (2), the linear fit. The experimental radius of gyration R_g_ is equal to 3.34±0.05 nm. The logarithm of the scattering intensity *I* is displayed as a function of the squared momentum transfer *s* = 4πsin*θ*/λ; λ is the X-ray wavelength (0.124 nm) and 2*θ* is the scattering angle.

**Supplementary References**

1 Kukic, P. *et al.* Protein Dielectric Constants Determined from NMR Chemical Shift Perturbations. *J. Am. Chem. Soc.* 135, 16968–16976 (2013).

2 Baker, N., Sept, D., Joseph, S., Holst, M. & McCammon, A. Electrostatics of nanosystems: Application to microtubules and the ribosome. *Proc. Natl. Acad. Sci. USA* 98 (2001).
